# Multifocal Glucocorticoid-Associated Osteonecrosis: Clinical Characteristics and Systemic Molecular Features

**DOI:** 10.3390/biomedicines14071463

**Published:** 2026-06-27

**Authors:** Kosuke Arita, Tomohiro Shimizu, Hotaka Ishizu, Yusuke Ohashi, Kentaro Homan, Daisuke Takahashi, Akihiro Ishizu, Norimasa Iwasaki

**Affiliations:** 1 Department of Orthopaedic Surgery, Faculty of Medicine and Graduate School of Medicine, Hokkaido University, Sapporo 060-8638, Japan; kou.dr.2415124@gmail.com (K.A.); k.houman@med.hokudai.ac.jp (K.H.);; 2 Department of Medical Laboratory Science, Faculty of Health Sciences, Hokkaido University, Sapporo 060-8638, Japan

**Keywords:** multifocal osteonecrosis, glucocorticoid-associated osteonecrosis, proteomic biomarkers, hypercoagulability, vascular dysfunction

## Abstract

**Background:** Multifocal osteonecrosis involving three or more anatomical sites is an uncommon but severe manifestation of glucocorticoid-associated osteonecrosis and may be associated with systemic clinical backgrounds. This study investigated the clinical characteristics and exploratory serum proteomic profiles of multifocal osteonecrosis using clinical and proteomic analyses. **Methods:** We analyzed 107 patients who underwent surgery for osteonecrosis of the femoral head between 2019 and 2024. Whole-body MRI was used to detect multifocal lesions. Patients were classified into glucocorticoid-related osteonecrosis of the femoral head (GO) and multifocal glucocorticoid-related osteonecrosis (MGO). Clinical variables were compared, and multivariate logistic regression identified clinical factors associated with multifocal osteonecrosis. Serum proteomic profiling using nanoLC–MS/MS was performed as an exploratory analysis to compare protein expression among GO, MGO, and osteoarthritis controls. **Results:** Multifocal osteonecrosis was identified in 31 patients (29.0%). Patients with MGO were younger (42.6 vs. 50.9 years, *p* = 0.021) and had higher glucocorticoid doses (59.5 vs. 48.5 mg, *p* = 0.005). Hematologic diseases (OR 14.51, 95% CI 3.42–86.69, *p* < 0.001) and skin manifestations (OR 3.17, 95% CI 1.05–10.44, *p* = 0.046) were independently associated with multifocal osteonecrosis. Exploratory proteomic analysis showed protein expression patterns related to fibrinolysis, coagulation, inflammation, and vascular homeostasis in MGO. **Conclusions:** Multifocal osteonecrosis was associated with systemic clinical backgrounds and showed exploratory vascular- or coagulation-related proteomic patterns within glucocorticoid-associated osteonecrosis.

## 1. Introduction

Osteonecrosis is characterized by the death of bone marrow and all cellular components within the affected bone, including osteocytes, adipocytes, and hematopoietic cells, due to an insufficient blood supply caused by various underlying mechanisms [1]. Osteonecrosis most frequently affects the femoral head (ONFH), with an estimated annual incidence of approximately 10,000–20,000 new cases in the United States and 2000–3000 in Japan [2], representing a considerable and increasing disease burden [3,4,5].

Multifocal osteonecrosis, defined as osteonecrosis occurring in three or more distinct anatomical sites, is a relatively rare condition, reported in approximately 3–11% of all osteonecrosis cases [6]. Recent studies utilizing whole-body MRI have revealed that asymptomatic osteonecrosis is more common than previously believed, with multifocal lesions occurring at a much higher rate (20–86%) than earlier estimates suggested [7,8,9]. The underlying conditions associated with multiple osteonecroses are diverse and include connective tissue diseases, hematological disorders, and respiratory infections [10,11,12,13]. A common feature among these conditions is a history of steroid use [9]; however, most existing studies and case reports have focused on single diseases, and there is no clear consensus on the role of steroid dosage or pulse therapy in osteonecrosis development. Although alcohol-associated osteonecrosis has been reported at two anatomical sites, reports on its involvement in multifocal osteonecrosis remain scarce [14,15,16].

These observations suggest that multifocal osteonecrosis, affecting three or more joints, may be associated with distinct clinical backgrounds and pathophysiological features compared with single-site glucocorticoid-associated osteonecrosis. Recent evidence has also indicated that multifocal osteonecrosis may be associated with hematologic abnormalities and systemic inflammatory manifestations, such as skin involvement, which may reflect or coexist with underlying vascular dysfunction and hypercoagulability. However, the systemic mechanisms underlying multifocal osteonecrosis remain poorly understood, and molecular evidence linking multifocal involvement to systemic vascular or coagulation-related alterations is limited. Therefore, the present study aimed to clarify the clinical characteristics and exploratory serum proteomic profiles of multifocal glucocorticoid-associated osteonecrosis by combining whole-body MRI-based phenotyping with serum proteomic profiling.

## 2. Materials and Methods

### 2.1. Study Design and Participants

This study is a prospective consecutive case series of patients who underwent surgery for osteonecrosis of the femoral head (ONFH) at Hokkaido University Hospital between April 2019 and December 2024. ONFH was diagnosed by two orthopedic surgeons (TS and DT) according to the diagnostic criteria established by Sugano et al. [17]. Patients with alcohol-induced ONFH who had not received glucocorticoid therapy were excluded from the analysis. This study was approved by the local ethics committee of Hokkaido University Hospital (approval number 019-0130), and informed consent was obtained from all participants.

Based on a previous study [9], a cohort of approximately 86 patients was considered sufficient to detect clinically meaningful differences between groups with adequate statistical power (power = 0.8, effect size = 0.8). The collected demographic and clinical data included sex; age at initial consultation; maximum daily glucocorticoid dose; history of glucocorticoid pulse therapy; alcohol consumption; smoking status; and comorbidities such as connective tissue disease, kidney disease, hematologic disease, and skin disorders. Alcohol abuse was defined as alcohol consumption exceeding 400 mL per week, which is a significant risk factor for ONFH [18]. A subgroup analysis excluding patients with hematologic diseases was performed to evaluate whether clinical differences between the GO and MGO groups persisted after removal of hematologic comorbidities.

### 2.2. Whole-Body MRI

Whole-body MRI (WB-MRI) was performed using a 1.5-T MRI system (Magnetom Avanto; Siemens Healthcare, Erlangen, Germany). T1-weighted (T1W) turbo spin-echo sequences (TR 600–800 ms; echo time 14 ms; slice thickness 6 mm; slice gap 12 mm; coronal Section 3 mm) were obtained from the neck to the ankles, with a total scan time of 20–25 min. Because osteonecrosis is typically asymptomatic before femoral head collapse and shows a characteristic band-like pattern on T1-weighted imaging, T1W imaging was selected for evaluation. Osteonecrosis becomes symptomatic after collapse due to bone marrow edema and joint effusion. WB-MRI was performed using T1W imaging alone, without STIR or T2-weighted fat-suppressed sequences. All WB-MRI scans were obtained during hospitalization under standardized imaging conditions. Multifocal osteonecrosis was defined as bone necrosis involving three or more distinct anatomical sites. Patients with glucocorticoid-induced ONFH without multifocal involvement were classified as the glucocorticoid-related ONFH group (GO), whereas those with glucocorticoid-induced multifocal osteonecrosis were classified as the multifocal glucocorticoid-related osteonecrosis group (MGO).

### 2.3. Proteome Analysis

To explore serum proteomic patterns potentially associated with multifocal osteonecrosis, proteomic analysis was performed in three groups: GO, MGO, and a control group consisting of patients with hip osteoarthritis (OA) who underwent surgery during the same period. Three independent serum samples per group were randomly selected after stratification by age and sex and pooled prior to exploratory shotgun proteomic profiling. This pooling strategy was used to identify global molecular trends rather than interindividual variability. The approach also enabled broad screening of serum proteins while partially reducing the influence of interindividual variability.

Serum samples (10 μL) were depleted of the six most abundant plasma proteins (albumin, IgG, IgA, transferrin, haptoglobin, and α1-antitrypsin) using the MARS Hu-6 spin cartridge (Agilent Technologies, Santa Clara, CA, USA) according to the manufacturer’s instructions. The depleted samples were concentrated to 50 μL using a 3 kDa cut-off ultrafiltration device (Merck Millipore, Burlington, CA, USA) and processed as follows: (1) reduction with 100 μL of 100 mM NH_4_HCO_3_ containing 0.15% DTT at 57 °C for 30 min; (2) alkylation with 100 μL of 100 mM NH_4_HCO_3_ containing 1% IAA at room temperature; (3) digestion with modified trypsin (50 mM NH_4_HCO_3_) overnight at 30 °C; and (4) peptide drying using a centrifugal concentrator (CC-105, TOMY SEIKO, Tokyo, Japan), followed by reconstitution in 0.1% formic acid (30 μL). After centrifugation (20,000× *g*, 10 min), the supernatant was subjected to nanoLC–MS/MS analysis.

Mass spectrometry was performed using an UltiMate 3000 nano-HPLC system (Dionex, Sunnyvale, CA, USA) coupled to a Q Exactive Plus mass spectrometer (Thermo Fisher Scientific, Waltham, MA, USA). Peptides were separated on an Aurora emitter column (IonOpticks, 25 cm × 75 μm, 1.6 μm C18). Spectra were acquired with Xcalibur software version 4.3 (Thermo Fisher Scientific, MA, USA), and data were analyzed using Mascot version 3.0 (Matrix Science, London, UK) against the SWISS-PROT Human database (Taxonomy ID: 9606). Search parameters were as follows: peptide tolerance 10 ppm; MS/MS tolerance 0.1 Da; one missed cleavage; fixed carbamidomethylation of cysteine; variable oxidation of methionine; ion score cutoff 10.

### 2.4. Statistical Analysis

Univariate analysis was conducted using chi-square or Fisher’s exact test for categorical variables and Student’s *t*-test or Mann–Whitney U test for continuous variables, as appropriate. Multivariate logistic regression analysis was performed to calculate the odds ratios and 95% confidence intervals (CIs) for the occurrence of multifocal osteonecrosis. Analyses were adjusted for significant background factors. All statistical analyses were conducted using GraphPad Prism 10 for macOS (version 10.4.1; GraphPad Software, Boston, MA, USA), with statistical significance set at *p* < 0.05. Because serum samples were pooled within each group, inferential statistical testing was not performed for the exploratory proteomic analysis.

## 3. Results

### 3.1. Patients and Their Characteristics

Between April 2019 and December 2024, 135 consecutive patients underwent surgery for ONFH at Hokkaido University Hospital. After excluding 28 patients with alcohol-induced ONFH who had not received glucocorticoid therapy, 107 patients were included in the final analysis. Their demographic and clinical characteristics are summarized in Table 1. The mean age at initial consultation was 49.0 years, and 86 patients (80.4%) had bilateral ONFH. Multifocal osteonecrosis, defined as involvement of three or more anatomical sites, was identified in 31 patients (29.0%). The mean maximum daily dose of glucocorticoids was 51.8 mg, and 46 patients (43.0%) received glucocorticoid pulse therapy. Comorbidities included connective tissue diseases in 51 patients (47.7%), kidney disease in 11 (10.3%), hematologic diseases in 15 (14.0%), and skin manifestations in 49 (45.8%). The hematologic diseases included acute lymphoblastic leukemia (ALL), idiopathic thrombocytopenic purpura (ITP), and malignant lymphoma. Skin manifestations included butterfly erythema in 14 patients and cutaneous graft-versus-host disease (GVHD) in 8, observed in patients with systemic lupus erythematosus (SLE) and post-transplantation status, respectively. Other manifestations included ITP-associated purpura in 3 patients, atopic dermatitis in 3, pemphigus vulgaris in 3, pemphigus foliaceus in 3, and other manifestations in 15, including those associated with dermatomyositis, polymyositis, and adult-onset Still’s disease.

### 3.2. Comparison of Demographic and Clinical Data Between Patients with and Without Multifocal Osteonecrosis

The demographic and clinical characteristics of patients with glucocorticoid-induced multifocal osteonecrosis (MGO) and those without multifocal involvement (GO) are presented in Table 2. The MGO group had a significantly younger mean age at initial consultation than the GO group (42.6 vs. 50.9 years, *p* = 0.021). The mean maximum daily glucocorticoid dose was also significantly higher in the MGO group (59.5 vs. 48.5 mg, *p* = 0.005). A markedly higher proportion of patients in the MGO group had hematologic diseases (38.7% vs. 4.0%; *p* < 0.001) and skin symptoms (71.0% vs. 35.6%; *p* < 0.001). These findings suggest that multifocal osteonecrosis was associated with younger age, higher maximum daily glucocorticoid dose, and systemic clinical backgrounds, including hematologic diseases and skin symptoms.

After excluding patients with hematologic diseases, 92 patients remained for subgroup analysis (Table S1). Among them, multifocal osteonecrosis was identified in 19 patients (20.7%). In this subgroup, bilateral ONFH, connective tissue disease, and skin symptoms were significantly more frequent in the MGO group than in the GO group (Table S2).

### 3.3. Multivariate Analysis of the Occurrence of Multifocal Osteonecrosis

Multivariate logistic regression analysis was conducted to identify independent clinical factors associated with multifocal osteonecrosis (Table 3). Hematologic disease showed the strongest association with multifocal osteonecrosis, with an odds ratio (OR) of 14.51 (95% confidence interval [CI]: 3.42–86.69, *p* < 0.001). Skin manifestations were also independently associated with multifocal osteonecrosis (OR = 3.17, 95% CI: 1.05–10.44, *p* = 0.046). These findings suggest that hematologic disease and skin manifestations are important systemic background factors associated with multifocal osteonecrosis.

### 3.4. Proteome Analysis

**Figure 1:**
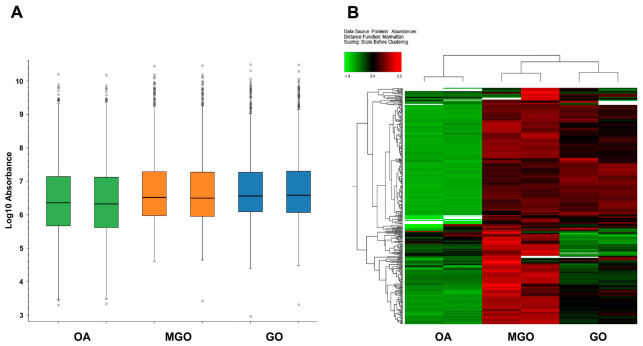
Proteome analysis. To identify serum proteomic patterns associated with multifocal osteonecrosis, preoperative serum samples pooled within each group were subjected to exploratory shotgun proteomic analysis. (**A**) The number of proteins identified in duplicate samples from each group. (**B**) Protein expression profiles across groups.

**Figure 2:**
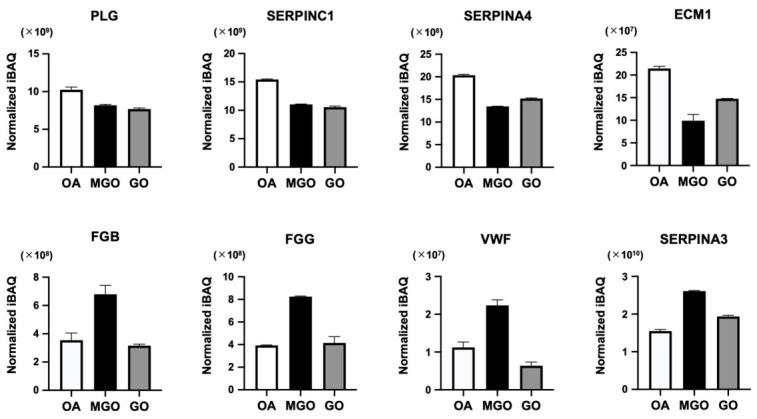
Quantitative Comparison of Protein Expression. Comparison of protein expression levels among OA (osteoarthritis patients), MGO (glucocorticoid-induced multifocal osteonecrosis patients), and GO (glucocorticoid-induced osteonecrosis of the femoral head [ONFH] without multifocal osteonecrosis patients). PLG, plasminogen; SERPINC1, antithrombin-III; SERPINA4, kallistatin; ECM1, extracellular matrix protein 1; FGB, fibrinogen beta chain; FGG, fibrinogen gamma chain; VWF, von Willebrand factor; SERPINA3, alpha-1-antichymotrypsin.

Serum proteomic profiling was performed to compare protein expression patterns among the GO, MGO, and osteoarthritis (OA) control groups. The total number of identified proteins was comparable across groups (Figure 1A), yet group-specific expression profiles were observed (Figure 1B). Further hierarchical clustering revealed visually distinct protein expression patterns between the GO and OA groups and, more prominently, between the MGO and GO groups (Figure 2).

Pathway enrichment analysis using the Reactome database suggested possible differences in coagulation-related pathways. Plasminogen (PLG) and antithrombin-III (SERPINC1) levels were lower in both the GO and MGO groups compared with OA controls, suggesting a possible reduction in fibrinolytic and anticoagulant capacity. Kallistatin (SERPINA4) and extracellular matrix protein 1 (ECM1) were further reduced in the MGO group relative to both GO and OA groups, suggesting a possible relationship with vascular dysfunction or reduced vascular protection. Conversely, fibrinogen β-chain (FGB), fibrinogen γ-chain (FGG), and von Willebrand factor (VWF) were higher in the MGO group, suggesting a proteomic pattern compatible with hypercoagulability and potential microthrombus-related processes. In addition, alpha-1-antichymotrypsin (SERPINA3), an acute-phase reactant, was higher in both GO and MGO groups compared with OA controls, with the highest levels observed in the MGO group. This pattern suggests that inflammatory, vascular, and coagulation-related alterations may be associated with multifocal osteonecrosis.

## 4. Discussion

The findings of this study suggest that multifocal osteonecrosis involving three or more joints is associated with distinct systemic clinical backgrounds within glucocorticoid-associated osteonecrosis. Compared with patients who had glucocorticoid-induced ONFH without multifocal involvement, those with multifocal osteonecrosis were significantly younger at diagnosis and had received higher maximum daily glucocorticoid doses. In addition, hematologic diseases and skin manifestations were identified as independent clinical factors associated with multifocal osteonecrosis, suggesting that systemic background factors may be related to multifocal lesion distribution, rather than indicating local joint susceptibility alone.

Exploratory proteomic analysis showed descriptive differences in serum protein expression patterns between the GO and MGO groups, particularly in proteins related to coagulation and vascular homeostasis. These findings provide supportive proteomic patterns that are consistent with vascular- and coagulation-related alterations in multifocal osteonecrosis and complement the observed clinical associations with hematologic disease and skin manifestations. Interestingly, the incidence of bilateral ONFH observed in this cohort (80.4%) was higher than previously reported in the literature. This may reflect greater overall glucocorticoid exposure in our surgical population or enhanced lesion detection through whole-body MRI screening, underscoring the potential underestimation of lesion multiplicity in standard clinical practice. The relatively high prevalence of multifocal osteonecrosis observed in this cohort may partly reflect the systematic use of whole-body MRI screening, which enables the detection of asymptomatic lesions that may remain unrecognized in routine clinical practice.

Although serum coagulation-related tests showed no significant differences among groups, exploratory proteomic analysis showed lower levels of key fibrinolytic and anticoagulant proteins, including PLG and SERPINC1, in patients with multifocal osteonecrosis. These proteins are essential for preventing excessive clot formation and maintaining adequate blood flow [19,20,21]. Their decreased levels suggest a possible reduction in fibrinolytic and anticoagulant capacity and a potential tendency toward microvascular thrombosis, consistent with previous studies implicating hypercoagulability in osteonecrosis pathogenesis [22,23,24]. Conversely, fibrinogen components (FGB and FGG) and VWF were higher in the MGO group compared with both the GO and OA groups, providing a proteomic pattern consistent with a hypercoagulable tendency in multifocal osteonecrosis. The increase in these proteins may be associated with impaired microcirculatory flow and thrombus formation [25,26], which may exacerbate bone ischemia and osteonecrosis [27,28]. These proteomic findings align with previous animal model studies; for instance, Ikemura et al. [29] demonstrated similar alterations in coagulation and fibrinolytic system proteins in a steroid-induced rabbit model of osteonecrosis. This concordance between human exploratory proteomic patterns and experimental data raises the possibility that hypercoagulability and impaired vascular integrity may be involved in glucocorticoid-associated multifocal osteonecrosis.

Hematologic diseases frequently present with abnormalities in coagulation- and fibrinolysis-related proteins, which may partly explain their association with multifocal osteonecrosis in this study. The subgroup analysis excluding patients with hematologic diseases suggested that multifocal involvement was not explained solely by hematologic diseases. Although hematologic disease was strongly associated with multifocal osteonecrosis in the main analysis, multifocal osteonecrosis was still observed after patients with hematologic diseases were excluded. In this subgroup, skin symptoms were more frequent in the MGO group than in the GO group, and connective tissue disease was also more frequent in the MGO group. These findings provide supportive clinical evidence that hematologic diseases alone may not fully explain multifocal lesion distribution. However, because the proteomic analysis was based on pooled serum samples, this subgroup analysis could not directly determine whether the observed protein expression patterns were independent of hematologic diseases.

In addition, because many conditions in the MGO group inherently promote hypercoagulability, the specific contribution of glucocorticoids to multifocal lesion distribution remains uncertain. It is possible that higher glucocorticoid doses reflect greater underlying disease severity necessitating treatment, rather than acting as a direct etiologic factor in multifocal osteonecrosis. Future studies should include control cohorts of glucocorticoid-exposed patients without prothrombotic comorbidities to further clarify this relationship. Skin manifestations were also significantly more common in patients with multifocal osteonecrosis than in those with osteonecrosis confined to the femoral head. Many of these manifestations, such as butterfly erythema and cutaneous graft-versus-host disease, are associated with systemic disorders involving vascular inflammation and endothelial dysfunction [30,31].

Exploratory proteomic analysis showed lower levels of ECM1 and SERPINA4 in the MGO group. ECM1 plays a critical role in extracellular matrix stability and vascular integrity [32], whereas SERPINA4 contributes to vascular regulation [33]. Additionally, SERPINA3 was higher in the multifocal osteonecrosis group compared with both the GO and OA groups. SERPINA3 is an acute-phase protein involved in inflammatory and tissue remodeling processes [34,35]. The combination of higher SERPINA3 and lower ECM1 and SERPINA4 suggests a proteomic pattern related to vascular dysfunction, altered vascular protection, and systemic inflammation in patients with multifocal osteonecrosis. These findings provide a biological context for the association between systemic vascular or inflammatory backgrounds and the skin manifestations observed in multifocal osteonecrosis. Given that butterfly erythema is a hallmark of SLE and that SLE severity can be associated with vascular involvement, cutaneous manifestations may be interpreted as indicators of systemic inflammatory or vascular backgrounds associated with multifocal osteonecrosis. This hypothesis warrants further investigation in prospective studies.

This study has several limitations. First, although a power analysis indicated a sufficient sample size, this was a single-center study, which may limit the generalizability of the findings. Multicenter studies are needed to validate these results in populations with diverse clinical backgrounds. The marked baseline imbalance between the GO and MGO groups may have acted as an important source of confounding; therefore, residual confounding cannot be excluded, and the observed clinical associations should be interpreted cautiously. The wide confidence intervals in the multivariable analysis, particularly for hematologic disease, reflect limited event numbers and reduced precision of the estimates. Second, our cohort comprised surgical cases only, which may have introduced selection bias by overrepresenting patients with more advanced ONFH, greater overall glucocorticoid exposure, or more severe systemic background diseases. Consequently, these findings may not be directly applicable to early or subclinical osteonecrosis. Third, the serum proteomic analysis was based on pooled samples within each group; therefore, interindividual variability could not be assessed. In addition, OA patients were selected as surgical controls without osteonecrosis for the proteomic analysis; however, OA itself may influence systemic inflammatory and protein expression profiles, and this should be considered when interpreting comparisons with the OA group. Independent validation using targeted methods, such as ELISA or PRM, was not performed. Thus, the proteomic findings should be interpreted as exploratory and require validation in larger independent cohorts using individual serum samples. Furthermore, because residual confounding related to underlying disease severity and disease-specific characteristics cannot be completely excluded, the observed proteomic differences should be interpreted as descriptive associations rather than disease-specific molecular signatures of multifocal osteonecrosis. Fourth, although whole-body MRI was used for comprehensive lesion detection, screening was performed using T1-weighted imaging alone, without STIR or T2-weighted fat-suppressed sequences. Therefore, small, early, or edema-dominant lesions may have been underdetected. Furthermore, because multifocal osteonecrosis was defined based on imaging findings, some lesions were clinically asymptomatic, and their clinical significance may differ from that of symptomatic multifocal disease. Finally, cumulative glucocorticoid dose, treatment duration, route of administration, and timing relative to osteonecrosis onset were not consistently available in this cohort. Therefore, total glucocorticoid exposure could not be fully assessed using maximum daily dose and pulse therapy history alone. In particular, the potential effect of long-term low-dose glucocorticoid therapy may have been underestimated.

## 5. Conclusions

This study suggests that multifocal osteonecrosis is associated with distinct systemic clinical backgrounds within glucocorticoid-associated osteonecrosis. Patients with multifocal osteonecrosis were younger, had higher maximum daily glucocorticoid doses, and showed a higher prevalence of hematologic diseases and skin manifestations, indicating an association with systemic clinical backgrounds. Exploratory proteomic analysis showed protein expression patterns related to coagulation, fibrinolysis, inflammation, and vascular homeostasis, providing hypothesis-generating support for the potential involvement of vascular- and coagulation-related alterations in multifocal osteonecrosis. These findings underscore the importance of recognizing multifocal osteonecrosis as a condition associated with systemic background factors and highlight the need for future studies focusing on validation using individual serum samples, functional analysis, risk stratification, and early detection strategies to improve patient outcomes and prevent disease progression.

## Figures and Tables

**Table 1:** Clinical characteristics of the participants.

	*N* = 107
Sex ratio, male:female	45:62
Mean age at initial consultation, years	49.0 (17.0)
Bilateral ONFH, cases	86 (80.4%)
Multifocal osteonecrosis, cases	31 (29.0%)
Maximum glucocorticoid dose, mg	51.8 (18.1)
Glucocorticoid pulse therapy, cases	46 (43.0%)
Drinking, cases	31 (29.0%)
Smoking, cases	37 (34.6%)
Connective tissue disease, cases	51 (47.7%)
Kidney disease, cases	11 (10.3%)
Hematologic disease, cases	15 (14.0%)
Skin symptoms, cases	49 (45.8%)
Butterfly erythema	14 (13.1%)
GVHD	8 (7.5%)
ITP-associated purpura	3 (2.8%)
Atopic dermatitis	3 (2.8%)
Pemphigus vulgaris	3 (2.8%)
Pemphigus foliaceus	3 (2.8%)
Others	15 (14.0%)

Data presented as mean (standard error of the mean) or percentage. ONFH, osteonecrosis of the femoral head.

**Table 2:** Univariate analysis of the GO group and the MGO group.

	GO Group *N* = 76	MGO Group *N* = 31	*p*-Value
Sex ratio, male:female	33:43	12:19	0.654
Mean age at initial consultation, years	50.9 (17.0)	42.6 (15.6)	0.021 *
Bilateral ONFH, cases	59 (77.6%)	27 (87.1%)	0.263
Maximum Glucocorticoid dose, mg	48.5 (16.6)	59.5 (19.5)	0.005 *
Glucocorticoid pulse therapy, cases	31 (40.8%)	15 (48.4%)	0.471
Drinking, cases	22 (28.9%)	9 (29.0%)	0.993
Smoking, cases	26 (34.2%)	11 (35.5%)	0.900
Connective tissue disease, cases	36 (47.4%)	14 (45.2%)	0.836
Kidney disease, cases	10 (13.2%)	1 (3.2%)	0.125
Hematologic disease, cases	3 (4.0%)	12 (38.7%)	<0.001 *
Skin symptoms, cases	27 (35.6%)	22 (71.0%)	<0.001 *
Serum concentration			
Total Cholesterol, mg/dL	196.4 (46.5)	201.0 (48.7)	0.655
HDL Cholesterol, mg/dL	62.7 (20.6)	67.1 (20.9)	0.327
LDL Cholesterol, mg/dL	111.7 (38.7)	106.4 (51.1)	0.811
Platelets, ×10^3^/μL	246.0 (88.2)	252.6 (97.9)	0.745
PT-INR	1.01 (0.25)	1.00 (0.14)	0.860
APTT, second	28.9 (4.48)	29.6 (5.06)	0.511

Data are presented as mean (standard error of the mean) or percentage. * *p* < 0.05. GO, glucocorticoid-induced osteonecrosis; MGO, glucocorticoid-induced multifocal osteonecrosis; HDL, high-density lipoprotein; LDL, low-density lipoprotein; PT-INR, prothrombin time-international normalized ratio; APTT, activated partial thromboplastin time.

**Table 3:** Multiple logistic regression analysis of the occurrence of multifocal osteonecrosis.

	Odds Ratio	95% Confidence Interval		*p*-Value
Sex, female	1.321	0.476	3.764	0.594
Mean age at initial consultation	0.982	0.950	1.014	0.277
Maximum Glucocorticoid dose	1.003	0.968	1.042	0.871
Bilateral ONFH	3.105	0.673	20.15	0.182
Hematologic disease	14.51	3.417	86.69	<0.001 *
Skin symptoms	3.171	1.046	10.44	0.046 *

* *p* < 0.05. ONFH, osteonecrosis of the femoral head.

## Data Availability

The data presented in this study are available from the corresponding author upon reasonable request.

## Supplementary Materials

The following supporting information can be downloaded at: https://www.mdpi.com/article/10.3390/biomedicines14071463/s1, Table S1. Clinical characteristics of the participants after excluding patients with hematologic diseases. Table S2. Univariate analysis of the GO and MGO groups after excluding patients with hematologic diseases.

## Abbreviations

The following abbreviations are used in this manuscript:

ONFH	Osteonecrosis of the Femoral Head
GO	Glucocorticoid-related Osteonecrosis of the Femoral Head
MGO	Multifocal Glucocorticoid-related Osteonecrosis
OA	Osteoarthritis
WB-MRI	Whole-body Magnetic Resonance Imaging
MRI	Magnetic Resonance Imaging
HDL	High-density Lipoprotein
LDL	Low-density Lipoprotein
PT-INR	Prothrombin Time-International Normalized Ratio
APTT	Activated Partial Thromboplastin Time
ALL	Acute Lymphoblastic Leukemia
ITP	Idiopathic Thrombocytopenic Purpura
GVHD	Graft-versus-host Disease
SLE	Systemic Lupus Erythematosus
