# Supplementary material for: Multifocal Glucocorticoid-Associated Osteonecrosis: Clinical Characteristics and Systemic Molecular Features
Source: Biomedicines. 2026 Jun 27;14(7):1463. doi: 10.3390/biomedicines14071463 (PMC13405343; doi:10.3390/biomedicines14071463)
Supplement: Supplementary file 1 [file biomedicines-14-01463-s001.zip › biomedicines-4380800-supplementary.pdf]

Supplementary Table S1. Clinical characteristics of the participants after excluding patients with hematologic diseases.

	N=92
Sex ratio, male:female	39:53
Mean age at initial consultation, years	49.7 (16.7)
Bilateral ONFH, cases	75 (81.5%)
Multifocal osteonecrosis, cases	19 (20.7%)
Maximum glucocorticoid dose, mg	49.2 (16.0)
Glucocorticoid pulse therapy, cases	41 (44.6%)
Drinking, cases	27 (29.3%)
Smoking, cases	32 (34.8%)
Connective tissue disease, cases	67 (72.8%)
Kidney disease, cases	11 (12.0%)
Skin symptoms, cases	39 (42.4%)

Data presented as mean (standard error of the mean) or percentage. ONFH, osteonecrosis of the femoral head.

Supplementary Table S2. Univariate analysis of the GO and MGO groups after excluding patients with hematologic diseases.

	GO group N=73	MGO group N=19	*P*-value
Sex ratio, male:female	33:40	6:13	0.284
Mean age at initial consultation, years	51.1 (16.8)	44.4 (15.4)	0.115
Bilateral ONFH, cases	57 (78.1%)	18 (94.7%)	0.001*
Maximum Glucocorticoid dose, mg	47.4 (17.7)	52.8 (12.3)	0.232
Glucocorticoid pulse therapy, cases	30 (41.1%)	11 (57.9%)	0.189
Drinking, cases	22 (30.1%)	5 (26.3%)	0.745
Smoking, cases	26 (35.6%)	6 (31.6%)	0.742
Connective tissue disease, cases	36 (49.3%)	15 (78.9%)	0.021*
Kidney disease, cases	10 (13.7%)	1 (5.3%)	0.313
Skin symptoms, cases	25 (34.2%)	14 (73.7%)	0.002*
Serum concentration			
Total Cholesterol, mg/dL	198.4 (45.1)	194.8 (42.4)	0.765
HDL Cholesterol, mg/dL	63.0 (20.1)	69.7 (19.9)	0.205
LDL Cholesterol, mg/dL	114.1 (40.2)	84.0 (20.9)	0.149
Platelets, $\times 10^3/\mu L$	250.1 (87.5)	273.4 (110.1)	0.338
PT-INR	1.01 (0.25)	1.01 (0.12)	0.931
APTT, second	28.8 (4.48)	30.1 (5.56)	0.294

Data are presented as mean (standard error of the mean) or percentage. * $p < 0.05$. GO, glucocorti-coid-induced osteonecrosis; MGO, glucocorticoid-induced multifocal osteonecrosis; HDL, high-density lipoprotein; LDL, low-density lipoprotein; PT-INR, prothrom-bin time-international normalized ratio; APTT, activated partial thromboplastin time.

 https://doi.org/10.3390/biomedicines14071463
